# High oncostatin M predicts lack of clinical remission for patients with inflammatory bowel disease on tumor necrosis factor α antagonists

**DOI:** 10.1038/s41598-022-05208-9

**Published:** 2022-01-24

**Authors:** Angela Guo, Cameron Ross, Nilesh Chande, Jamie Gregor, Terry Ponich, Reena Khanna, Michael Sey, Melanie Beaton, Brian Yan, Richard B. Kim, Aze Wilson

**Affiliations:** 1 Department of Physiology and Pharmacology, Western University, Medical Sciences Building, Rm 216, London, ON N6A 5C1 Canada; 2 Division of Clinical Pharmacology, Department of Medicine, Western University, 339 Windermere Road, London, ON N6A 5A5 Canada; 3 Division of Gastroenterology, Department of Medicine, Western University, 339 Windermere Rd, London, ON N6A 5A5 Canada

**Keywords:** Predictive markers, Inflammatory bowel disease, Biomarkers, Gastroenterology

## Abstract

The interleukin-6 family cytokine, oncostatin-M (OSM) has been associated with response to tumor necrosis factor-α antagonists (anti-TNFs) in small cohorts of patients with inflammatory bowel disease (IBD). We aimed to evaluate the association between plasma OSM concentrations and response to anti-TNFs (infliximab and adalimumab) in both ulcerative colitis (UC) and Crohn’s disease (CD). A retrospective cohort study was conducted in patients with IBD with a history of anti-TNF exposure. Blood samples, collected prior to anti-TNF exposure, were analyzed by enzyme-linked immunosorbent assay for the presence and quantity of OSM. Clinical remission was assessed at 1-year post anti-TNF exposure in addition to the occurrence of surgery, hospitalization, corticosteroid use, and adverse drug events. Lastly the threshold OSM plasma concentration associated with anti-TNF non-response was assessed by receiver operator characteristic (ROC) curve analysis. Patients with IBD (CD, n = 82; UC, n = 40) were assessed. In both UC and CD, mean pre-treatment OSM concentrations were significantly lower in those who achieved clinical remission at 1-year (p < 0.0001). A threshold plasma OSM concentration of 168.7 pg/ml and 233.6 pg/ml respectively separated those who achieved clinical remission at 1-year on an anti-TNF from those who did not in CD and UC respectively (CD: area under the receiver operator characteristic curve, AUROC = 0.880, 95% CI 0.79–0.96; UC: AUROC = 0.938, 95% CI 0.87–1.00). High OSM concentrations were associated with anti-TNF discontinuation and use of rescue steroids in CD and UC. High pre-treatment OSM concentrations identify IBD patients at-risk of anti-TNF non-response at 1-year as well as other deleterious clinical outcomes.

## Introduction

The use of the tumor necrosis factor-α antagonists (anti-TNFs), infliximab and adalimumab, for the management of inflammatory bowel disease (IBD) continues to be refined. Since the approval for their use in both ulcerative colitis (UC) and Crohn’s disease (CD), a vast number of studies have been published to delineate their role in a variety of disease phenotypes including fistulizing disease, fulminant colitis and various extra-intestinal disease manifestations^1–3^. There is also an increasing awareness of the intricacies of their pharmacokinetic and pharmacodynamic profiles that have often led to unpredictable and sometimes suboptimal disease response^4,5^. Rates of response can range from 30 to 50% across various IBD study cohorts and "real-world" populations^4,6^. One of the main knowledge gaps in IBD therapeutics is the inability to predict which drug will be most effective for which patient. This has led to an iterative approach that results in patients having multiple IBD drug exposures over their lifetime^7,8^. This leaves individuals with IBD vulnerable to drug side effects, may delay the definitive management of their IBD and is associated with significant costs^9^. Currently, international guidelines recommend that anti-TNFs be implemented as first-line therapy followed by the anti-integrin vedolizumab in moderate to severe disease^10,11^. This does not reflect other options that are now approved, soon-to-be approved or the inter-individual differences in patients and their disease that may affect drug response. The importance of selecting the right biologic for a patient upfront is further reinforced by the fact that the highest chance for remission is seen with the first biologic and falls dramatically with exposure to subsequent agents^12^. Head-to-head trials of biologics are slowly emerging that may allow clinicians to assess the value of one biologic over another in various patient scenarios^13^. These will be a long time in coming and the sequencing of biologics in a patient’s treatment plan is a well-established problem today. This has led to calls for biomarkers that can guide decision-making for biologic selection^14^.

Recently, the interleukin (IL)-6-family cytokine oncostatin-M (OSM) has been suggested as a possible novel biomarker of anti-TNF response. OSM is a pleiotropic cytokine produced by hematopoietic cells, particularly type 1 T-helper (Th1) cells^15^. It has been identified as playing a role in multiple homeostatic processes including hepatic regeneration and bone metabolism. Conversely, it has been linked to several pathological processes including cancer and several diseases of chronic inflammation^15,16^. More recently, West *et.al.* (2017) identified that the expression of OSM in the intestinal stroma was correlated with the presence and severity of intestinal inflammation in IBD versus healthy controls^16^. They additionally noted that the intestinal expression of OSM was associated with poor response to anti-TNF therapy (infliximab, golimumab) in four small UC cohorts derived from trial datasets. They found that complete mucosal healing following infliximab therapy was achieved by 85% of patients with low OSM expression, but was observed in only 10–15% of those with high OSM expression. Given that the analysis of intestinal biopsies samples may not be feasible in a clinical setting, two groups completed small studies in 45 adult and 40 pediatric patients with CD respectively and similarly found that the pre-infliximab blood concentration of OSM was higher in patients who lost response to infliximab during the maintenance phase of treatment^17,18^. However, a recent study by Verstockt *et.al.* (2021) did not find a correlation between serum OSM and anti-TNF response^19^.

These mixed, but intriguing, data increase the need to further explore the utility of OSM as a clinically-actionable tool for the identification of those who would derive the most benefit from anti-TNF therapy. We aimed to evaluate the association between plasma OSM concentrations and the achievement of clinical remission on anti-TNFs (infliximab and adalimumab) in addition to other important clinical outcomes in both UC and CD, including the use of rescue glucocorticoid therapy, treatment discontinuation, surgical intervention and hospitalization.

## Methods

### Participants and procedures

A retrospective cohort study was carried out in participants with either CD or UC. All participants were seen between 2012 and 2019 as part of the Personalized Medicine Program at the London Health Sciences Centre (LHSC), a tertiary care centre affiliated with Western University (London, Canada). Included participants were adults greater than 17 years of age with an endoscopic and histological diagnosis of one of CD or UC who received treatment with an anti-TNF agent. All participants were required to have provided a blood sample within 4 weeks prior to anti-TNF exposure and were previously naïve to treatment with an anti-TNF agent. Eligible participants received either infliximab or adalimumab at the discretion of their treating gastroenterologist as per standard dosing guidelines (infliximab 5 mg/kg at 0, 2, 6 weeks followed by a per 8-week dosing schedule; adalimumab 160 mg, 80 mg, 40 mg induction strategy per 2-week interval followed by 40 mg per 2 weeks). Escalation of anti-TNF therapy during the study period was permitted and at the discretion of the treating physician. Participants were required to have at least one therapeutic infliximab concentration, defined as greater than or equal to 3 µg/ml or adalimumab concentration, defined as greater than 7.5 µg/ml demonstrated during the maintenance phase of therapy. The use of concomitant therapies such as glucocorticoids, 5-aminosalicylates, and/or one of methotrexate or azathioprine was also permitted and at the discretion of the treating physician. Participants were excluded if they were younger than 18 years of age, had prior exposure to an anti-TNF prior to the defined study period, had a sub-therapeutic infliximab or adalimumab concentration in the presence or absence of anti-drug antibodies during the follow-up period, or if there were missing data pertaining to their clinical response to the anti-TNF agent.

The baseline data collected on all participants included age, sex, weight, disease type (CD or UC), smoking history, disease duration (years since initial diagnosis to time of blood sample collection), disease location, anti-TNF received (infliximab or adalimumab) and all other IBD drug exposures.

Following inclusion, participants were monitored for up to one year following the commencement of the anti-TNF or until discontinuation of anti-TNF therapy. Participants were assessed for the primary outcome: the presence of clinical remission based on the Harvey Bradshaw Index (HBI) for CD (remission, HBI < 5) and the partial Mayo score for UC (remission, partial Mayo score < 2) at 52 weeks from initiation of the anti-TNF or at the time of anti-TNF discontinuation. Additionally, participants were assessed for secondary outcomes including the occurrence of surgery, hospitalization, adverse drug events (ADEs) attributed to anti-TNF therapy, anti-TNF discontinuation, and corticosteroid use during the 1-year follow-up period.

### Ethical considerations

The study protocol was approved by the Western University Health Sciences Research Ethics Board in accordance with the Tri-Council Policy Statement and all participants provided written, informed consent.

### Plasma OSM quantification

Blood samples collected prior to anti-TNF exposure were collected and plasma aliquots were extracted via centrifugation and stored at -80^•^C until further use. A commercial enzyme-linked immunosorbent assay (ELISA) was used for the colorimetric detection and quantification of human OSM as per the manufacturer’s protocol (human OSM ELISA kit, Thermo Fischer, Waltham, USA). The lower limit of detection was 1.37 pg/mL with concentrations below this threshold reported as a 0 value. Standard curves and patient samples were plated in duplicates.

### Statistical analyses

All statistical analyses were performed independently for patients with CD or UC using the GraphPad Prism 9 software (GraphPad Software Inc., San Diego, California) and R 4.0.3. A p value < 0.05 was considered significant.

Descriptive statistics were used to summarize data for all participants divided by the presence or absence of clinical remission at 1-year. Descriptive statistics included frequency distributions for categorical variables and medians with interquartile ranges (IQR) or ranges for continuous variables. Categorical variables were analyzed using a Fisher’s exact test. The Shapiro–Wilk test was used to test the normality of distribution for plasma OSM concentrations in all cohorts. Based on this, a Mann–Whitney U test was selected to compare the mean OSM concentrations between participants based on clinical remission at 1-year.

A receiver operating characteristic (ROC) and Youden index analysis^20^ were used to determine the threshold OSM plasma concentration associated with clinical remission at 1-year on anti-TNF therapy. Area under the ROC curve, in addition to the sensitivity and specificity, for the optimal thresholds are reported.

Secondary outcome analyses using Fisher’s exact test were performed to determine whether the occurrence of surgery, hospitalization, ADEs, anti-TNF-α discontinuation, or corticosteroid use was associated with OSM concentrations above or below the calculated cut-off threshold value.

Lastly, a multivariable modified Poisson regression was used to assess the impact of OSM concentration on the odds of achieving clinical disease remission at 1-year on anti-TNF therapy adjusting for the covariates age and sex. Additional covariates were selected by performing a univariate analysis. Covariates with a p value < 0.2 were included in the final model.

## Results

**Figure 1:**
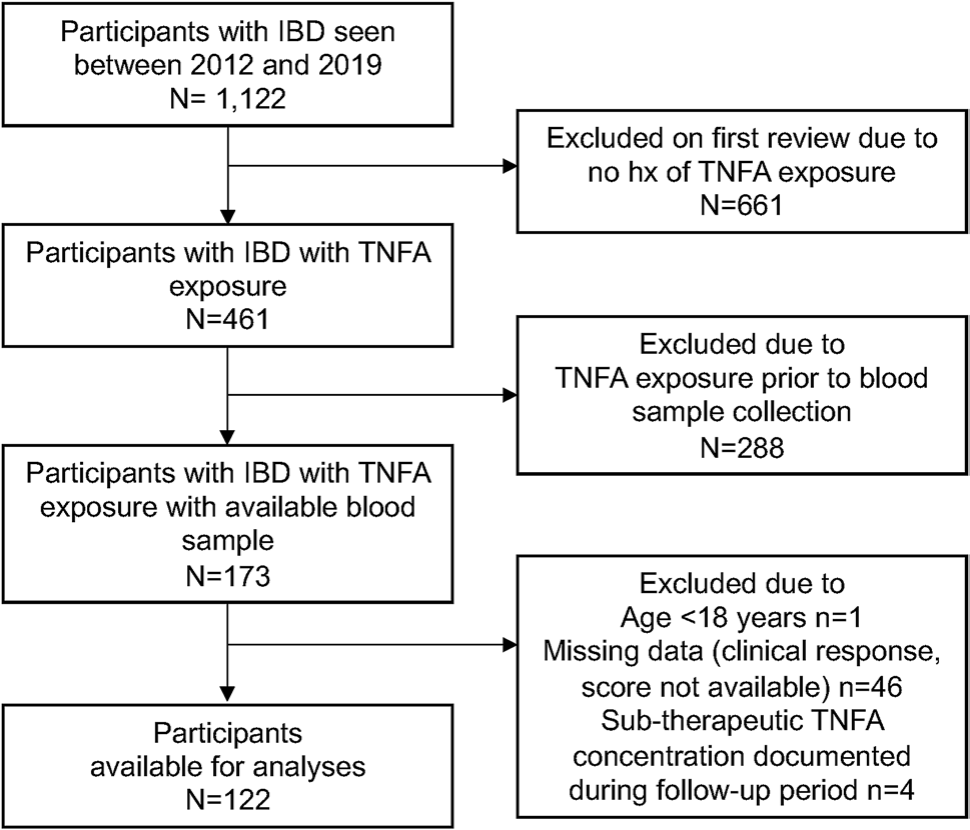
Study flowchart.

Participant selection is summarized in Fig. 1. Baseline characteristics by disease type and by disease remission status are summarized in Tables 1 and 2 respectively. A total of 1022 participants with IBD seen as part of the Personalized Medicine Program at LHSC were screened for inclusion. Of these individuals, 122 participants were included in the final analyses (CD, n = 82; UC, n = 40).

**Table 1:** Demographic characteristics by disease type.

Variable	Crohn’s disease (n = 82)	Ulcerative colitis (n = 40)
Age, years, (mean, range)	43.01 (18–78)	39.43 (21–73)
Female sex (%)	44 (53.7)	19 (47.5)
Weight, kg (mean ± SD)	81.27 ± 21.82	77.97 ± 16.06
Ileal (%)	25 (30.5)	–
Ileo-colonic (%)	44 (53.7)	–
Colonic (%)	10 (12.2)	–
Upper GI tract	3 (3.7)	–
Pan-colitis (%)	–	28 (70.0)
Left-sided colitis (%)	–	11 (27.5)
Proctitis (%)	–	1 (2.5)
Previous IBD-related surgery (%)	27 (32.9)	0 (0.0)
Smoking history (%)	18 (21.9)	5 (12.5)
Glucocorticoid exposure (%)	20 (24.4)	11 (27.5)
Infliximab exposure (%)	38 (46.3)	28 (70.0)
Adalimumab exposure (%)	44 (53.7)	12 (30.0)
Combination therapy with AZA (%)	35 (42.7)	16 (40.0)
Combination therapy with MTX (%)	14 (17.1)	3 (7.5)
Disease duration (mean ± SD)	10.94 ± 11.34	7.61 ± 8.79

Not significant, ns; kilogram, kg; standard deviation, SD; gastrointestinal, GI; inflammatory bowel disease, IBD; azathioprine, AZA; methotrexate, MTX.

**Table 2:** Demographic characteristics by disease activity.

Variable	Clinical remission (n = 78)	Clinically active Disease (n = 44)	p value
Age, years, (mean, range)	42.05 (18–78)	41.79 (18–65)	ns
Female sex (%)	43 (55.1)	19 (43.2)	ns
Weight, kg (mean ± SD)	81.99 ± 21.03	77.48 ± 18.58	ns
Crohn’s disease (%)	54 (69.2)	28 (63.6)	ns
Ileal (%)	20 (37.0)	3 (10.7)	0.01
Ileo-colonic (%)	27 (50.0)	17 (60.7)	ns
Colonic (%)	6 (11.1)	4(14.3)	ns
Upper GI tract	0 (0.0)	3 (10.7)	0.04
Baseline median HBI (IQR)	7 (2)	8 (3)	ns
Ulcerative colitis (%)	24 (30.8)	16 (36.4)	ns
Pan-colitis (%)	15 (60.0)	11 (68.8)	ns
Left-sided colitis (%)	9 (40.0)	4 (25.0)	ns
Proctitis (%)	0 (0)	1 (6.3)	ns
Baseline median partial Mayo score (IQR)	8 (1)	8 (3)	ns
Smoking history (%)	17 (21.8)	6 (13.6)	ns
Infliximab exposure (%)	45 (57.7)	23 (52.3)	ns
Adalimumab exposure (%)	33 (42.3)	21 (47.7)	ns
Combination therapy (%)	53 (67.9)	13 (29.5)	< 0.0001
Disease duration (mean ± SD)	8.70 ± 9.71	12.04 ± 12.29	ns

Not significant, ns; kilogram, kg; standard deviation, SD; gastrointestinal, GI; Harvey Bradshaw Index, HBI; interquartile range, IQR.

The baseline characteristics were similar for those achieving clinical remission on an anti-TNF versus those who did not with only a few exceptions (Table 2). Differences that were noted included individuals with CD who achieved clinical remission were more likely to have ileal disease than those who did not (37% versus 10%, p = 0.01). More participants with CD with active disease at 1-year had CD of the upper gastrointestinal tract (10% versus 0%, p = 0.04). Additionally, the use of combination therapy was more commonly reported in individuals who achieved clinical remission on an anti-TNF versus those who did not (67.9% versus 29.5%, p < 0.0001). The median follow-up for the total cohort was 12 months (IQR,0). Adverse events attributed to anti-TNF therapy occurred in 23 participants and included the following: infection (n = 19), psoriaform rash (n = 2), headache (n = 2).

**Figure 2:**
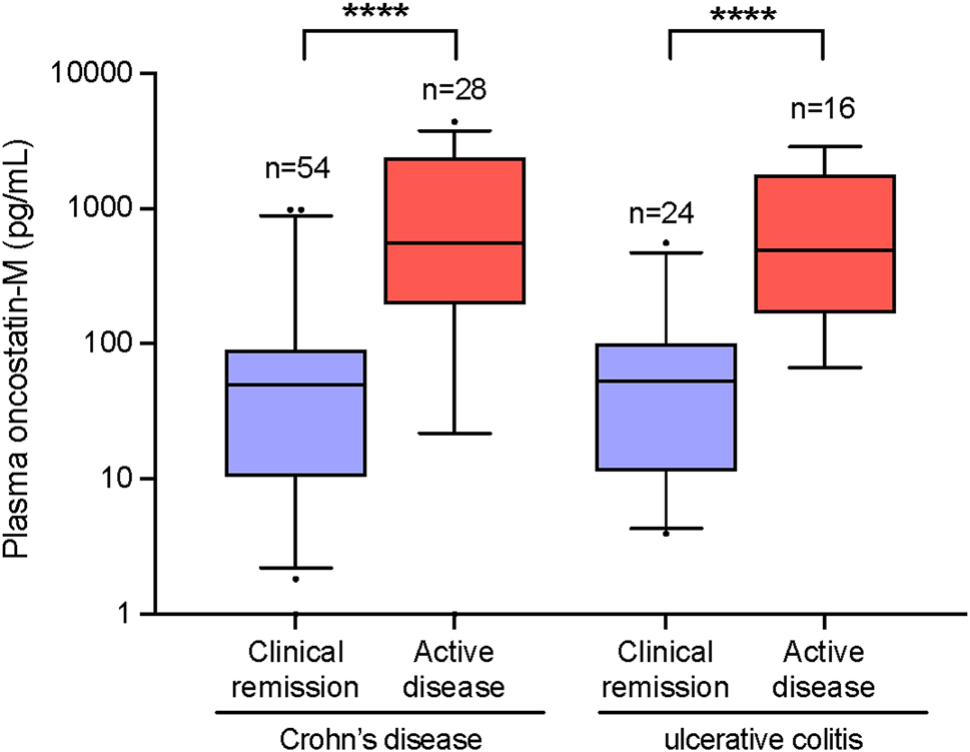
Mean plasma OSM concentrations in participants with Crohn’s disease and ulcerative colitis stratified by the presence or absence of clinical remission on a TNFA at 1-year are represented by box and whisker plot. Median values (thick horizontal line), 25th and 75th percentile values (box outline), 5–95% confidence intervals (whiskers); *p < 0.0001.

All participants had at least one documented infliximab trough concentration greater than 5 µg/ml during the assessment period. No participants had an infliximab trough concentration less than 5 µg/ml during the assessment period. Overall, mean plasma OSM concentrations were significantly higher in patients with CD or UC who did not achieve remission 1-year after receiving an anti-TNF (Fig. 2). For those with UC, 16 participants failed to achieve clinical remission at 1-year with a mean OSM concentration of 996.0 ± 986.1 pg/ml, while 24 participants achieved clinical remission at 1-year based on the partial Mayo score with a mean OSM concentration of 84.5 ± 119.7 pg/ml while (p < 0.0001). For those with CD, 28 participants failed to achieve clinical remission at 1-year with a mean OSM concentration of 1284.0 ± 1258.0 pg/ml, while 54 achieved clinical remission at 1-year based on the HBI with a mean OSM concentration of 110.3 ± 217.3 pg/ml while (p < 0.0001).

**Figure 3:**
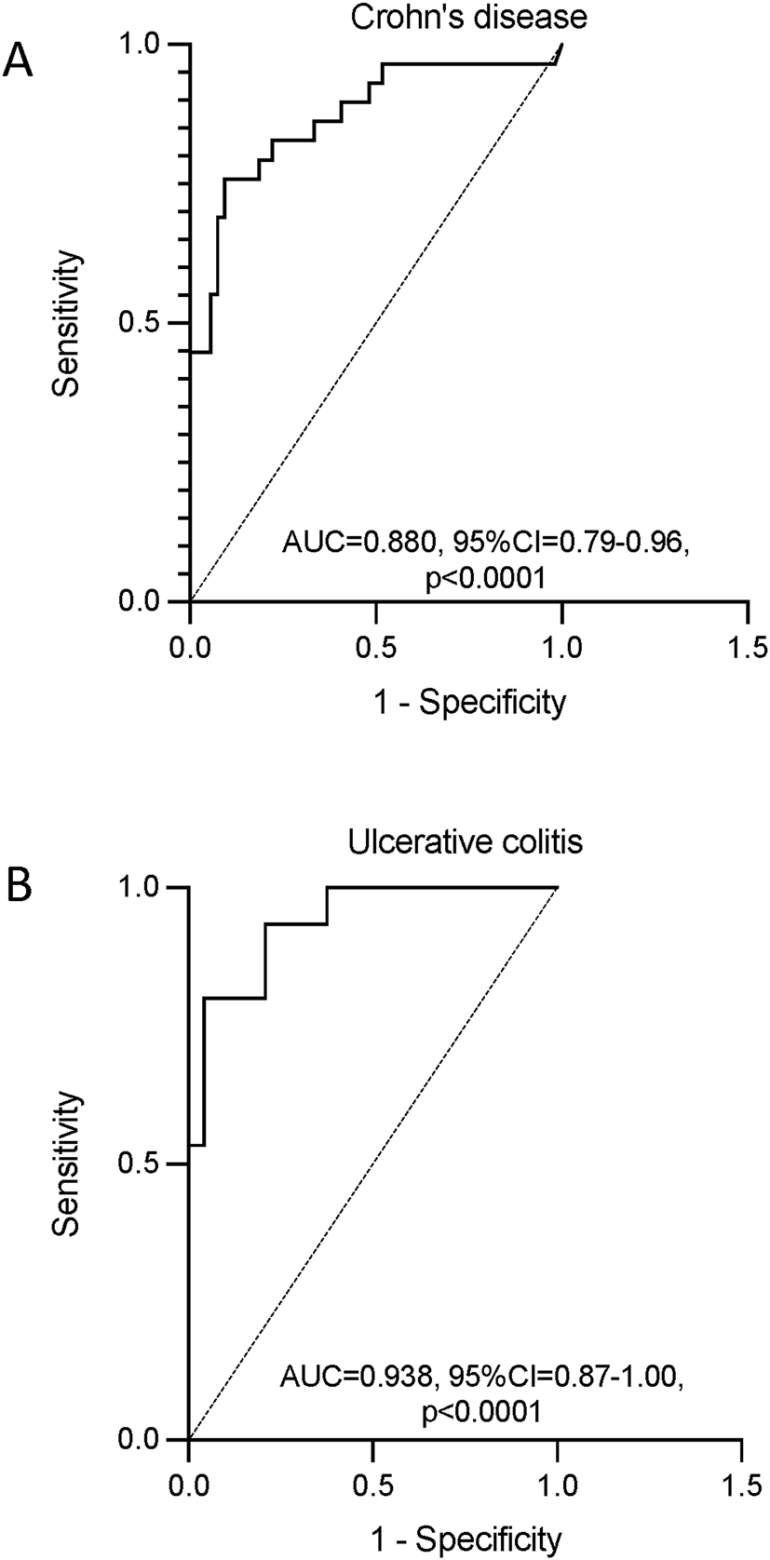
Receiver operator characteristic analysis for mean plasma OSM concentrations for CD (**A**) and UC (**B**) participants with and without clinical remission at 1 year on anti-TNF therapy (**B**). Oncostatin-M, OSM; Crohn’s disease, CD; ulcerative colitis, UC; area under the curve (AUC); confidence interval (CI).

For participants with CD, a plasma OSM concentration of 168.7 pg/ml (area under the receiver operator characteristic curve, AUROC = 0.880, 95% CI 0.79–0.96) separated those who achieved clinical remission at 1-year on an anti-TNF from those who did not with a sensitivity (95% CI) of 76% (58–88%) and specificity of 91% (80–96%) (Fig. 3A). For participants with UC, a plasma OSM concentration of 233.6 pg/ml (AUROC = 0.938, 95% CI 0.87–1.00) separated those who achieved clinical remission at 1-year on an anti-TNF from those who did not with a sensitivity of 80% (55–93%) and specificity of 96% (79–99%) (Fig. 3B).

Furthermore, in UC, participants with a plasma OSM concentration above the threshold concentration of 233.6 pg/ml were more likely to discontinue their anti-TNF prior to 1-year (OR 10.71, 95% CI 1.81–56.7, p = 0.0085) and require rescue corticosteroids (OR 6.71, 95% CI 1.58–24.9, p = 0.02). No difference was seen in the occurrence of hospitalization (OR 3.56, 95% CI 0.79–15.8, p = 0.19), surgery (OR 7.80, 95% CI 0.99–104.3, p = 0.09), and anti-TNF-related adverse drug events (OR 1.05, 95% CI 0.18–5.3, p = 0.99) (Table 3).

**Table 3:** Incidence of secondary outcomes in participants with ulcerative colitis.

Variable	Above threshold OSM concentration*	Below threshold OSM concentration*	Odds ratio (95% CI)	P value
Discontinuation of anti-TNF (%)	6/13 (46.2)	2/27 (7.4)	10.71 (1.81–56.7)	0.0085
Hospitalization (%)	4/13 (30.8)	3/27 (11.1)	3.56 (0.79–15.8)	0.19
Use of rescue glucocorticoids (%)	7/13 (53.8)	4/27 (14.8)	6.71 (1.58–24.9)	0.02
Surgery (%)	3/13 (23.1)	1/27 (3.7)	7.80 (0.99–104.3)	0.09
Anti-TNF-related ADE (%)	2/13 (15.4)	4/27 (14.8)	1.05 (0.18–5.3)	0.99

Oncostatin-M, OSM; confidence interval, CI; tumor necrosis factor-α, TNF; adverse drug event.

*Threshold OSM concentration in ulcerative colitis is defined as 233.6 pg/ml.

In CD, participants with a plasma OSM concentration above the threshold concentration of 168.7 pg/ml, were more likely to discontinue their anti-TNF at 1-year (OR 7.75, 95% CI 2.50–21.7, p = 0.0002), and require rescue corticosteroid therapy (OR 8.00, 95% CI 2.42–26.3, p = 0.0002). No difference was seen in the frequency of hospitalization, surgical intervention or anti-TNF-related adverse event (Table 4).

**Table 4:** Incidence of secondary outcomes in participants with Crohn’s disease.

Variable	Above threshold OSM concentration*	Below threshold OSM concentration*	Odds ratio (95% CI)	P value
Discontinuation of anti-TNF (%)	15/28 (53.6)	7/54 (12.9)	7.75 (2.50–21.7)	0.0002
Hospitalization (%)	8/28 (28.6)	8/54 (14.8)	2.30 (0.77–6.49)	0.15
Use of rescue glucocorticoids (%)	14/28 (50.0)	6/54 (11.1)	8.00 (2.42–26.3)	0.0002
Surgery (%)	7/28 (25.0)	5/54 (9.2)	3.27 (0.95–10.4)	0.09
Anti-TNF-related ADE (%)	8/28 (28.6)	9/54 (16.7)	2.00 (0.62- 6.08)	0.25

Oncostatin-M, OSM; confidence interval, CI; tumor necrosis factor-α, TNF; adverse drug event.

*Threshold OSM concentration in Crohn’s disease is defined as 168.7 pg/ml.

On multi-variable analysis adjusting for the covariates age, sex, disease type, disease duration, use of glucocorticoids or combination therapy, every 10 pg/mL increase in OSM concentration resulted in a 4% decrease in the likelihood of achieving clinical remission at 1 year on anti-TNF Therapy (Table 5).

**Table 5:** Multiple variable regression for the effect on clinical remission at 1-year on anti-TNF therapy.

Variable	Risk ratio (95% CI)	Multivariate *p*	Univariate *p*
OSM (pg/ml)	0.996 (0.994–0.998)	0.002	< 0.0001
Age (years)	0.975 (0.935–1.017)	0.24	0.98
Male sex	1.964 (0.603–6.734)	0.27	0.26
Combination therapy	0.151 (0.039–0.495)	0.0031	< 0.0001
Glucocorticoid use	8.013 (2.157–33.55)	0.0026	< 0.0001
Disease duration (years)	0.958 (0.908–1.01)	0.11	0.19
Disease type (CD)	0.770 (0.219–2.803)	0.68	< 0.0001

Oncostatin-M, OSM; picogram, pg; kilogram, kg; Crohn’s disease, CD; confidence interval, CI; tumor necrosis factor-α, TNF.

## Discussion

Our study demonstrates that high plasma concentrations of the IL-6 family cytokine, OSM are associated with poor therapeutic outcomes to anti-TNF therapy in both CD and UC. Specifically, plasma OSM concentrations were significantly higher in participants with UC and CD who did *not* achieve clinical remission at 1-year compared to participants who did (Fig. 2). Plasma OSM concentrations above the threshold of 168.7 pg/mL and 233.6 pg/mL in CD and UC, respectively were associated with failure to achieve clinical remission at 1 year. The multivariable regression analysis showed that plasma OSM, irrespective of age, sex, disease sub-type, disease duration, receipt of glucocorticoids or combination therapy, was an independent predictor of clinical remission. Incremental increases in plasma OSM were associated with a decreasing likelihood of clinical remission.

These findings are consistent with studies that reported associations between the expression of OSM in the intestinal tissues or blood and anti-TNF failure over time^16–19^. West *et.al.* (2017) reported a higher mucosal expression of OSM in UC patients with anti-TNF failure while Bertani *et.al.* (2020) found that pre-treatment serum OSM concentrations were significantly higher in CD patients who did not achieve clinical or endoscopic remission at 1-year following treatment with infliximab^16,17^. Additionally, Minar *et.al.* (2019) demonstrated that high plasma-based OSM in pediatric patients with CD was associated with poor biochemical and clinical response to infliximab at 1-year^18^. Moreover, the threshold plasma OSM concentration separating remission from non-remission in CD reported by Minar *et.al.* (2019) was highly similar to the threshold defined herein (143.5 pg/mL versus 168.7 pg/mL). Interestingly, Verstockt *et.al.* (2021) found no significant association between serum OSM concentrations and endoscopic remission despite reporting a significantly higher mucosal OSM gene expression in IBD patients who did not achieve endoscopic remission after initiating therapy with an anti-TNF^19^. This contradictory finding may relate to differences in study methodology including the reporting of a relative, rather than whole, OSM measurement as well as the timing of assessment of the primary outcome. All studies evaluating remission (biochemical, mucosal, and clinical) with an anti-TNF at 1-year reported a statistically significant difference in blood-based OSM concentrations between those achieving remission versus those who did not^17,18^. Verstockt *et.al.* (2021) evaluated remission at 6 months which may have been a too-short time interval^19^. This idea is supported by the work of Minar *et.al.* (2019) where anti-TNF-mediated biochemical and clinical remission at 3 months were not associated with plasma OSM concentrations; however, anti-TNF-mediated biochemical and clinical remission at 1 year were associated with plasma OSM concentrations less than 143.5 pg/mL^18^.

Despite the association of OSM and anti-TNF failure, its role in the mechanisms of IBD and anti-TNF response remain unclear. West et al. (2017) demonstrated that OSM gene expression is highly enriched in patients with IBD, particularly in those resistant to anti-TNF therapy. They proposed that OSM synergizes with TNF in the intestine to enhance the expression of pro-inflammatory genes and that neutralization of OSM could plausibly dampen anti-TNF resistance^16^. Verstockt et al. (2019) also suggested that OSM may serve as a future novel target in IBD therapeutics; however, they also surmised that as a biomarker, OSM may represent a more medically-refractory disease phenotype based on their own original work^19,21^. Conversely, rather than acting synergistically, OSM and TNF-α cytokines may represent the activation of independent pro-inflammatory pathways. OSM is known to induce signaling via the JAK-STAT pathway^15,22^. This revelation, in addition to the success of janus kinase inhibitors in anti-TNF-naïve as well as anti-TNF-resistant disease, may indirectly highlight activation of a non-TNF dominant pro-inflammatory immune cascade^23^. Overall, further study is needed.

We additionally evaluated the association between high OSM plasma concentrations and relevant clinical outcomes such as the occurrence of surgery, hospitalization, anti-TNF-related ADEs, anti-TNF discontinuation, and the need for rescue corticosteroids. In the CD and UC cohorts respectively, there was no significant association between high plasma OSM defined as above the threshold of 168.7 pg/mL (CD) or 233.6 pg/ml (UC) and the occurrence of surgery, hospitalization or anti-TNF-related ADEs (Tables 3 and 4). However, both CD and UC patients were more likely to discontinue their anti-TNF-α drug and/or require rescue corticosteroids (Tables 3 and 4). Interestingly, a higher proportion of participants went on to surgery in the high OSM groups; however, this did not achieve statistical significance. This could be a consequence of a relatively small sample size and could be further explored in a larger, prospective cohort. Overall, these findings further highlight the clinical utility of OSM for potentially reducing other deleterious outcomes associated with anti-TNF failure.

Limitations of this study include its retrospective design and lack of data assessing endoscopic and histologic remission. The latter is balanced by the reporting of additional outcomes (steroid use, treatment cessation) that are highly relevant to patient care. Additionally, differences in OSM thresholds between UC and CD are not known, but should be further investigated in future mechanistic studies. Strengths of this study are its longitudinal and real-world data collection. Data are thus more generalizable and applicable to clinical populations.

## Conclusion

Ultimately, plasma OSM concentrations were significantly higher in patients who did not achieve remission 1-year after initiating anti-TNF therapy. High OSM concentrations were associated with anti-TNF discontinuation and increased use of corticosteroids in both UC and CD. These findings provide further support that OSM may represent an important biomarker of anti-TNF response. Further study is needed in larger, prospective cohorts and the mechanistic underpinnings linking OSM and anti-TNF response need to be defined.
